# Notes and News

**Published:** 1904-05-28

**Authors:** 


### NOTES AND NEWS.

H.R.H. the Prince of Wales will preside at the 146th anniversary festival of the Orphan Working School, on June 22nd.

There are three vacancies on the Council of the Royal College of Surgeons of England this year, and Mr. F. S. Eve, Mr. Bland Sutton, and Mr. Gilbert Barling are amongst the candidates in July.

Miss Dillwyn has made a most generous offer in connection with the Convalescent Home for the Swansea Hospital, in which she proposes to double any sum received by her for the Convalescent Fund during the present week.

We regret to state that Sir Henry Burdett met with a serious accident on Monday last when driving in the vicinity of Thrapston, Northamptonshire. The horse in the carriage he occupied was frightened by a motor, and overturned the vehicle, throwing out all the occupants Sir Henry Burdett received somewhat severe injuries to shoulder, chest, and thigh, besides being generally considerably bruised.

An extraordinary proceeding has come to light at the East Lothian Hospital. It was announced at the last meeting of the Haddington Town Council that it had become the custom at the hospital to open the letters addressed to patients and read them. It was stated that the letters were opened on the authority of the medical superintendent. The matter was referred to the hospital committee.

The editors of the boys' paper who published a recipe consisting of a mixture of nitric acid and mercury as a plating for bicycle handles deserve much censure. One fatality has already resulted. It is true that the recipe pointed out that the mixture should not be corked, but this is just a point which would easily escape attention, and anyone not knowing the nature of the mixture might insert a cork, thinking that its omission was an act of carelessness. In the case in question, the chemist himself corked the bottle containing the mixture, which exploded and caused the death of the boy purchaser. We sincerely hope that this accident may be made widely known, and prevent the further use of the dangerous recipe.

The latest site for a consumption sanatorium is to be the fiords of Greenland, where it is stated that a Washington medical man is going to conduct a hospital ship. The idea is the outcome of the physician's experience on an Arctic expedition, when it was observed that a consumptive benefited in a remarkable manner.

At the annual meeting of the University of Wales, the question as to a petition for a supplemental charter was raised to enable the University to grant degrees in medicine, surgery, and obstetrics. It was announced that the scheme had the approval of the Chancellor, the Prince of Wales, and a resolution to proceed with the charter was passed.

When Parliament assembles on Tuesday, Mr. Robert Cameron will ask the Home Secretary whether his attention has been called to the report of the Lister Institute of Preventive Medicine, "in which it is admitted that the injection of antitoxin causes a large number of rashes, inflammation of joints—ascribed to something inherent in the serum of the horses or other animals—and also an increased number of cases of paralysis;" and "whether, in view of this statement, he will consider the advisability of taking steps to prohibit the manufacture of serum in laboratories licensed under the Vivisection Acts."

Last week Her Highness Princess Louise Augusta of Schleswig-Holstein, a Lady Vice-President of the South Kensington District of the League of Mercy, attended a Garden Party at Lowther Lodge, Kensington Gore, given by Dora, Countess of Chesterfield, Lady President of the District, by the kind permission of the Hon. Mr. and Mrs. William Lowther. Dr. Norman Hay Forbes, a Vice-President of the Tonbridge District, delivered a short address explaining the work of the League of Mercy, and a most enjoyable afternoon was spent in the garden. A selection of music was played by the Fleur De Lys Amateur Band. Lady Farquhar, Lady Gore, General Clifford, and Colonel Sir James Gildea were among those present. The League of Mercy is making considerable progress with communities of working people. Sir Henry Burdett gave an address at Harrod's Stores to the employés there a few weeks ago, which has resulted in a contribution of £156, and much more will probably be received within the present year. On Friday (May 20th) Sir Henry went to the Millbank estate of the London County Council, which the King and Queen visited privately in February last year. The model buildings consist of upwards of 2,000 rooms, representing 900 tenements, and the inhabitants consist largely of policemen, postmen, and members of the better artisan class. Sir Henry addressed a crowded meeting in the school-room on the estate, explaining the work, and the paramount necessity to all classes, of the hospital and its claim upon all the citizens. The meeting was enthusiastic, and it is hoped that the majority of the families residing at Millbank will become members of the League of Mercy. Mr. R. W. Granville Smith, J.P., presided, and in the course of the evening Sir Henry Burdett presented a number of certificates to members of the League working under Mr. W. J. Berry, a Vice-President of the Westminster District.

